# Accumulation of PHA in the Microalgae *Scenedesmus* sp. under Nutrient-Deficient Conditions

**DOI:** 10.3390/polym13010131

**Published:** 2020-12-30

**Authors:** Gabriela García, Juan Eduardo Sosa-Hernández, Laura Isabel Rodas-Zuluaga, Carlos Castillo-Zacarías, Hafiz Iqbal, Roberto Parra-Saldívar

**Affiliations:** Tecnologico de Monterrey, Escuela de Ingenieria y Ciencias, Campus Monterrey, Ave. Eugenio Garza Sada 2501, Monterrey 64849, Nuevo Leon, Mexico; a00828119@itesm.mx (G.G.); eduardo.sosa@tec.mx (J.E.S.-H.); laura.rodas@tec.mx (L.I.R.-Z.); carloscastilloz@tec.mx (C.C.-Z.)

**Keywords:** polyhydroxyalkanoates, *Scenedesmus* sp., microalgae, taguchi design, nutrients impact, carbon source

## Abstract

Traditional plastics have undoubted utility and convenience for everyday life; but when they are derived from petroleum and are non-biodegradable, they contribute to two major crises today’s world is facing: fossil resources depletion and environmental degradation. Polyhydroxyalkanoates are a promising alternative to replace them, being biodegradable and suitable for a wide variety of applications. This biopolymer accumulates as energy and carbon storage material in various microorganisms, including microalgae. This study investigated the influence of glucose, N, P, Fe, and salinity over the production of polyhydroxyalkanoate (PHA) by *Scenedesmus* sp., a freshwater microalga strain not previously explored for this purpose. To assess the effect of the variables, a fractional Taguchi experimental design involving 16 experimental runs was planned and executed. Biopolymer was obtained in all the experiments in a wide range of concentrations (0.83–29.92%, *w*/*w* DW), and identified as polyhydroxybutyrate (PHB) by FTIR analysis. The statistical analysis of the response was carried out using Minitab 16, where phosphorus, glucose, and iron were identified as significant factors, together with the P-Fe and glucose-N interactions. The presence of other relevant macromolecules was also quantified. Doing this, this work contributes to the understanding of the critical factors that control PHA production and present *Scenedesmus* sp. as a promising species to produce bio-resources in commercial systems.

## 1. Introduction

Plastics are essential materials in modern everyday life [1], but the unsustainable nature of the petroleum from which plastics originate and the environmental damage caused by their accumulation in marine and terrestrial deposits has triggered the search for environmentally friendly substitutes [2], such as bioplastics [3]. One type of biopolymer that has generated great interest in recent years is polyhydroxyalkanoates (PHAs) [4,5,6]. PHAs are natural carbon and energy storage compounds present in many photosynthetic organisms, synthesized in response to nutrient deficiency conditions in the presence of a carbon source [7]. They have great durability, biodegradability, biocompatibility, and properties similar to conventional thermoplastics [8]. Being derived from renewable biological materials, these biopolymers constitute a promising alternative to solve the environmental problems caused by petrochemical plastics [9].

Thanks to their unique combination of properties, PHAs have the potential to be used in a wide range of applications [10]. Their biodegradability and biocompatibility allow their use in medical applications [11], where they can be used in the manufacture of implants [12,13], wound dressings [13,14], or drug delivery carriers [13,15,16,17]. These applications require a product with high purity, so the carbon source from which they are originated must not be contaminated [5]. When waste streams are used as a substrate, the obtained PHA can be directed to other sectors. Their thermal processability makes them ideal for the fabrication of environmentally friendly packaging [18,19,20]. Furthermore, their blend-ability and mechanical properties make them very useful for the materials and nanotechnology sectors [5,21], where they can be used in the fabrication of compostable batteries [22], films [23], nanoparticles [24], and nanocomposites [25,26].

PHA accumulation occurs naturally in photosynthetic organisms, such as microalgae [27]. Microalgae are known for their great potential to produce metabolites like proteins, lipids, carbohydrates, and pigments, which can be transformed into products for the food, pharmaceutical, and medical industries [28], or for the generation of green energy [29]. Additionally, they play a fundamental role in wastewater remediation [30,31,32,33] and carbon dioxide sequestration [34,35,36,37]. Microalgae can use waste streams to obtain the nitrogen (N) and phosphorus (P) they need for growth [38,39], or they can use them as carbon sources for the production of polyhydroxyalkanoates [40,41]. This characteristic, coupled with their rapid growth, their low space and water requirements, and their use of sunlight as an energy source, makes them an economically attractive alternative as PHA-producing organisms [42].

**Figure 1:**
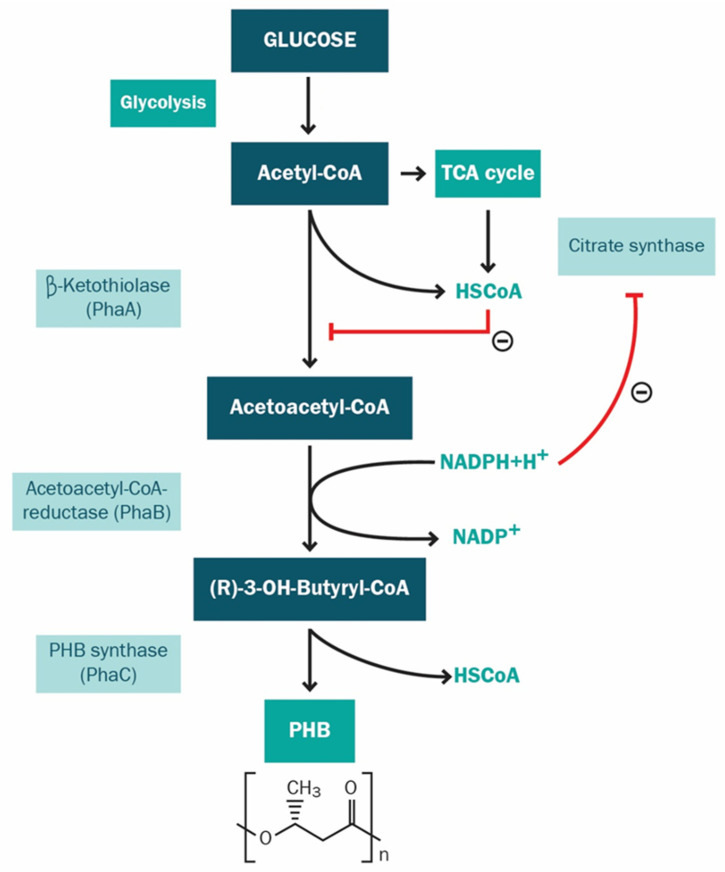
Polyhydroxybutyrate (PHB) synthesis pathway from acetyl-CoA and some of their related regulatory circuits. Under starvation conditions (nitrogen and phosphorus deficiency), overall reduction in the rate of protein synthesis is observed. The electron transfer activity is downregulated and ATP production decreases, resulting in an increased NADPH pool that favors the accumulation of the storage compound PHB. However, the synthesis of certain proteins required for acclimation process is enhanced upon nutrient limitation. Iron presence is required for the upregulation of at least one of these proteins (alkaline phosphatase enzyme). PHB precursors in bold in dark boxes. Related metabolic processes in bold in medium-color boxes. Critical enzymes in light-color boxes. Red lines indicate negative regulatory effects. **Abbreviations: Acetil-CoA** Acetil coenzima A, **TCA cycle** Citric acid cycle, **HSCoA** Coenzyme A, **NADPH+H^+^** Reduced nicotinamide adenine dinucleotide phosphate, **NADP^+^** Nicotinamide adenine dinucleotide phosphate, **ATP** Adenosine triphosphate.

The synthesis of PHA in photosynthetic microorganisms starts with the consumption of acetyl-CoA (Figure 1). Two acetyl-CoA molecules are joined together to form one acetoacetyl-CoA molecule in a β-ketothiolase (PhaA) catalyzed condensation reaction. This molecule is then reduced to D-3-hydroxybutyryl-CoA by nicotinamide adenine dinucleotide phosphate (NADPH)-dependent acetoacetyl-CoA reductase (PhaB). Finally, PHB synthase (PhaC) catalyzes the binding of D-3-hydroxybutyryl to an existing polyhydroxybutyrate (PHB) molecule through an ester bond, releasing CoA [43]. The chemical composition of the resulting PHA polymers can be manipulated by varying the substrates fed to the producer organism [44]. Since the synthesis of PHA is regulated at the enzymatic level [7], the intracellular concentration of acetyl-CoA and free CoA plays a central role in the synthesis of the polymer. The enzymatic activity and the availability of the PHA precursors are dependent on the presence of different compounds in the medium [45].

Various studies suggest that there are important interrelations between the PHA biosynthetic pathway and those of the central carbon metabolism [46], especially the glycogen pathway, one of the major cellular carbohydrate forms in cyanobacteria, which is degraded to a simpler compound: glucose. Dutt and Srivastava [47] showed that in PHA accumulating microalga photosynthetically grown under N-depletion, up to 87% of the carbon in PHB is derived from intracellular carbon reserves rather than from the CO_2_ fixed during the cultivation, but it was not clear which specific metabolic routes related with carbon fixation provided precursors for PHA synthesis. The work done by Koch et al. [48] revealed that products of glycogen degradation could be key precursors for PHA production, which indicated that the biosynthetic pathways for PHA and carbohydrates accumulation do not compete which each other, but are interconnected.

Although PHAs are naturally present in microalgae, their percentage content by weight is usually relatively low (generally below ten percent of the cell biomass) compared to that of other microorganisms such as bacteria [49,50]. A large screening study in which 137 different strains were analyzed for their PHA production reported that under normal growth conditions natural concentration of the biopolymer was lower than 3.5% *w*/*w* DW on 133 of the studied strains [51]. Some other studies have reported PHA concentrations below nine percent of the dry weight for liter. However, under suitable culture conditions, this concentration can be significantly increased [52]. Access to sufficient light and carbon, favorable pH and salinity values, and an adequate concentration of nutrients in the growing medium are some of the factors that can potentially improve PHA production [53]. The identity and values of these factors vary between different strains of microalgae. Nitrogen and phosphorus deficiency is a commonly used strategy to increase PHA accumulation in microalgae since these two nutrients are crucial for algae development [49,52,54]. However, some other metals commonly added to microalgae cultures could play a role in biopolymer production [55]. Among them, iron could be of interest since it is closely related to the growth, metabolism, and photosynthetic activity of microalgae [56,57]. Although some works have explored the influence of this metal in the production of biomass [58], lipids [59], carbohydrates, and proteins [60] by microalgae, its impact on PHA production has remained largely unexplored. Ultimately, the physiology of each strain and the environmental conditions to which it is adapted, confer them different sensitivity to nutrient limitation [61] and resistance to environmental and nutritional stress, which determines its PHA producing capacities as a response to said constraints [62].

The successful use of microalgae for the production of biopolymers requires identifying the most promising species, considering the environmental and nutritional factors that influence production metabolism. In the present work, the production of PHA by *Scenedesmus* sp., a microalgae strain that had not been previously explored for this purpose, was studied. This strain is commonly found in fresh and brackish waters [63] in many regions around the world [64] like North America [65], China [66], Thailand [67], among others. Factors that could potentially impact biopolymer accumulation were identified and their influence was evaluated using a fractional factorial Taguchi experimental design (described in further in Section 2.2). The polymer obtained was extracted, quantified, and characterized by FT-IR spectrometry. The presence of lipids and carbohydrates, two other macromolecules considered of interest, was also quantified. It is expected that this work will contribute to the integral utilization of microalgae for the production of bioplastic in commercial systems.

## 2. Materials and Methods

### 2.1. Microalgae Strain and Culture Medium

All experiments were carried out with biomass from *Scenedesmus* sp. (UTEX 1589) obtained from the UTEX culture collection center of the University of Texas. Initially, biomass was cultured autotrophically in a 2-L Erlenmeyer flask using BG-11 medium and cold white fluorescent lamps (100 μmol m^−2^ s^−1^) at 20 °C, in a greenhouse (to avoid contamination) located in the laboratories of the Center for Studies for Sustainable Development of ITESM (Monterrey, Mexico; 25°39′13.6″ N 100°17′33.1″ W).

Later, *Scenedesmus* sp. was grown in BG-11 medium. Sixteen different modified culture media were prepared to have diverse nutrient conditions. For limited nitrogen conditions, the BG-11 medium without sodium nitrate (NaNO_3_) was prepared [68]. Additionally, the phosphorus content was limited by omitting dipotassium phosphate (K_2_HPO) from the preparation and the iron content by omitting ferric ammonium citrate ((NH_4_)_5_[Fe(C_6_H_4_O_7_)_2_]) from the preparation [49]. Half of the cultures were supplemented with 4 g L^−1^ of glucose as an additional carbon source and the rest with 1 g L^−1^. Finally, the salt concentration was varied by adding 0.5 or 2 g L^−1^ of NaCl. The pH of the media was adjusted to 8.2. All solutions were autoclaved.

### 2.2. Experimental Design

The first phase of the experiment was to identify the relevant factors that influence the production of bioplastic by the microalgae, that is, those that critically affect PHA production performance. Based on previous literature, five main factors were identified as potentially influential: glucose, nitrogen, phosphorus, iron, and salinity concentration. Next, an experimental Taguchi matrix was designed. The Taguchi method uses orthogonal arrays, which stipulate the way of conducting the minimal number of experiments that will give the information of all the factors that affect the performance parameter [69], in this case PHA PRA production. and the data analysis procedure was identified using MiniTab16. In total, sixteen experiments were performed with orthogonal arrangements in random order. Using this design, five factors were tested with two different experimental levels (low and high). Table 1 lists the low and high values assigned to each factor. These values were selected from data available in the literature [49,53,68,70,71]. Light intensity, working volume, CO_2_ concentration, and temperature were considered constant, the last two factors corresponding to ambient values.

### 2.3. Culture Conditions

Cells kept in 2 L of protein medium were used as inoculum (20 mL) for liquid cultures (131.25 mg L^−1^). The experiments were carried out for 14 days in 500-mL Erlenmeyer flasks containing 300 mL of BG-11 medium at 25 °C under continuous illumination with cold white light fluorescent lamps (100 μmol m^−2^ s^−1^) and atmospheric CO_2_ (200 mL s^−1^). Samples were taken for determination of chemical oxygen demand (COD), pH, volatile fatty acid content, biomass, and salinity every three days and for lipids and carbohydrates on days 0, 7, and 14. All experiments were performed in duplicate. At the end of the cultivation, the bioplastic production was analyzed.

### 2.4. Analytic Methods

For each of the analysis, a volume of 10 mL of the algae culture was taken and centrifuged (Thermo Scientific^TM^ 50126393, 4000 rpm, 15 min). The centrifuged sample was used as described.

#### 2.4.1. Biomass

Cell concentration was monitored by measuring the dry weight of the biomass [72,73]. 

Biomass productivity $P_{X}$ was calculated as shown in Equation (1), where $X_{t}$ is the biomass concentration (g L^−1^) at time t (days), and $X_{0}$ is the biomass concentration (g L^−1^) at time $t_{0}$.

(1) $$P_{X}=\;X_{t}-X_{0}/t-t_{0}$$

#### 2.4.2. COD

For determination of the chemical oxygen demand, 2 mL of the supernatant were placed in 10-mL COD vials (Hach^®^ 2125815), these vials were kept in a COD digester reactor for 2 h at 150 °C. After the completion of the reaction, the samples were cooled for 20 min inside the reactor and then stirred again to allow them to cool to room temperature until reaching 20 °C. Their optical density was read on a Hach spectrophotometer that directly gave the COD value of each sample.

#### 2.4.3. VFAs

For the determination of volatile fatty acids (VFA), 0.4 mL of 10% sulfuric acid were placed in Hach vials for VFA analysis (TNT 872, Hach^®^) with ethanediol, mixed by inversion and 0.4 mL of the culture supernatant was added, mixing again. The vials were kept in a digester reactor for 10 min at 100 °C, then allowed to cool to room temperature to approximately 20 °C. Following this, 0.4 mL of hydroxylammonium chloride, 0.4 mL of sodium hydroxide, and 2 mL of sulfuric acid was then added, mixing after the addition of each reagent. The sample stood for 3 min and its optical density was read on a Hach spectrophotometer that directly returned the VFA content.

#### 2.4.4. Total Lipid Content

The total lipid content was quantified using a colorimetric method [74]. The supernatant of the centrifuged sample was removed, and the pellet resuspended in 10 mL of bidistilled water. Subsequently, 100 µL of the sample was placed in a glass tube and 2 mL of concentrated sulfuric acid were added. Samples were incubated at 100 °C for 10 min and then placed for five minutes in an ice bath. Finally, 5 mL of phosphovanillin were added and incubated for 15 min at 37 °C. The absorbance was read at 530 nm using bidistilled water as a blank and the readings were compared against a calibration curve performed using commercial canola oil to obtain the total lipid content in mg mL^−1^.

#### 2.4.5. Total Carbohydrate Content

The total carbohydrate content was quantified using a colorimetric method [75]. The supernatant of the centrifuged sample was removed, and the pellet resuspended in 10 mL of bidistilled water. Subsequently, 200 µL of the sample was placed in a 2-mL Eppendorf tube and 200 µL of 5% phenol solution was added. After mixing for 5 s, immediately 1 mL of concentrated sulfuric acid was added, and the sample was stirred for 5 s again. The samples were incubated for 30 min at room temperature and the optical density at 488 nm was read using bidistilled water as a blank. The readings were compared against a calibration curve made using glucose, and the total carbohydrate content in mg/mL was obtained. All experiments were performed in duplicate. Average results are presented in the results section.

### 2.5. PHA Extraction and Quantification

The extraction method was based on the widely accepted chloroform extraction proposed by [76]. To extract the PHA from microalgae, 100 mL of the algae culture were taken and centrifuged (4700 rpm, 7 min), the pellet was collected and dried overnight in a drying oven at 45 °C, in Falcon tubes. The dry weight of the tubes was taken to obtain the dry weight of the biomass. Subsequently, the algae cells were washed with 5 mL of ethanol, then centrifuged (3000 g, 30 min, 10 °C) and the supernatant was discarded. The cells were resuspended in 10 mL of 4% commercial sodium hypochlorite solution and incubated for one hour at 37 °C. The solution was centrifuged (3000 g, 30 min, 10 °C), the supernatant was discarded and two more washes were carried out, with 5 mL of bidistilled water and 5 mL of ethanol, centrifuging after each one with the conditions previously used and collecting the pellet. The pellet was then transferred to glass tubes previously brought to constant weight and the polymers were dissolved in 10 mL of chloroform brought to the boiling point. This chloroform solution was passed through fiberglass filters (pore size 0.45 μm) and the chloroform was evaporated on a rotary evaporator at 45 °C. Finally, the dry weight of the polymers was taken and used to determine the total production of PHA.

Each biopolymer sample obtained after extraction was analyzed separately to determine the presence of polyhydroxyalkanoates. Analysis of the dry polymer was carried out with a Spectrum One FTIR infrared spectrometry kit (PerkinElmer Inc., Waltham, MA, USA), in the band of 400–4000 cm^−1^.

## 3. Results

### 3.1. Growth and Characterization of Biomass

**Figure 2:**
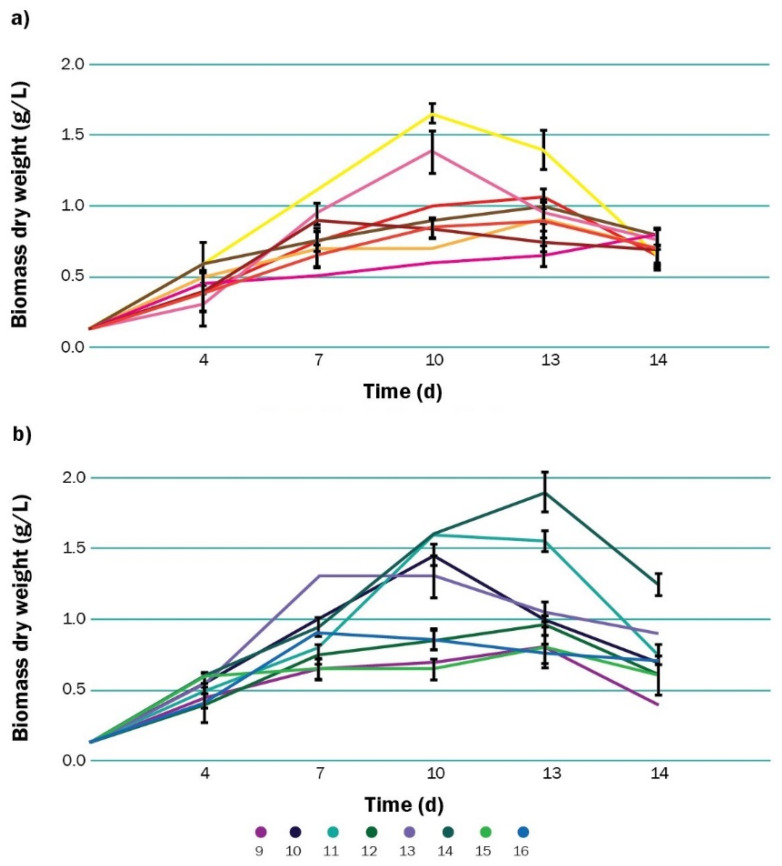
Biomass growth (dry weight) of *Scenedesmus* sp. on the 14 days-long experiment (**a**) under autotrophic growth (**b**) under heterotrophic growth. In each case, 20 mL of *Scenedesmus* cells (131.25 mg L^−1^) were inoculated in 300 mL and cultured for 14 days on modified BG-11 at 25 °C under continuous illumination (100 μmol m^−2^ s^−1^) and atmospheric CO_2_ pumping (200 mL s^−1^) without mechanical agitation. In (**a**), culture media was supplemented with 1 g L^−1^ of glucose. In (**b**), culture media was supplemented with 4 g L^−1^ of glucose.

The biomass growth during the cultivation of *Scenedesmus* sp. under mixotrophic conditions is shown in Figure 2 separated in two sets. For the sixteen experiments, the cultures did not present the lag phase of adaptation, instead of beginning in the exponential phase. Cell growth of microalgae was similar for experiments 1, 3, 4, 8, 9, 12, 14, and 15, which showed a maximum biomass concentration around day 13. For experiments 2, 5, 10, 11, and 16, this maximum measurement was obtained on day 10. Cultures 7 and 13 had their biggest biomass count on days 7 and 10. After these periods, cell growth stopped or entered the decay phase for all the experiments, except for number 6, which had not reached the stationary growth phase on day 14.

Experiment 13 was the fastest-growing culture, reaching their maximum biomass productivity of 0.167 g L^−1^d^−1^ on day 7. This was the highest value obtained for *Scenedesmus* across all the experiments. Experiment 14 presented the highest average biomass productivity, with 0.1194 g L^−1^d^−1^. This was the culture with heterotrophic growth, phosphate deficiency, and high salinity. There was a 55% drop from this value to the lowest average productivity obtained, which was 0.0534 g L^−1^d^−1^ for experiment 6. This was the culture with low glucose, phosphorus, and iron in combination with low salinity.

Three different behaviors were observed on the pH profile of the experiments, all of which started at a value of 8.2. Experiments 9, 11, and 12 presented a suit decrement to 4 on pH value at day 4 and remained around that value through all the cultivation time. Cultures 5, 10, and 13 showed a gradual reduction in pH that went from 8.2 to around 5 on day 14. The rest of the cultures went through an initial pH reduction but gradually recovered to reach a value near to 8 on day 13. After day 13, pH started decaying again on these cultures.

### 3.2. Analysis of Bioplastic Production

PHA presence was detected on the sixteen experiments performed. Experiment 6 presented the highest concentration of this storage compound with 29.92% *w*/*w* DW. The lowest concentration was detected on experiment 13, with a value of 0.83% *w*/*w* DW. Experiment 2 resulted in the highest PHA yield with a value of 0.171 g/L.

**Figure 3:**
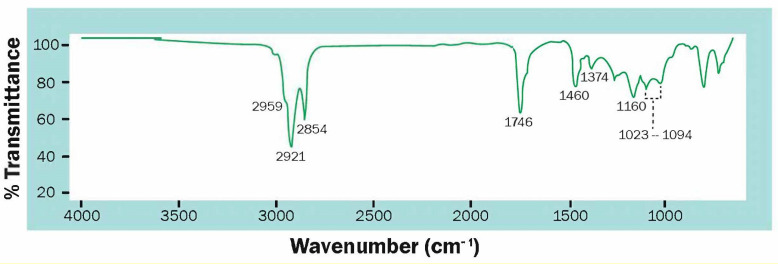
FT-IR spectrum of isolated PHB from experiment 1 with *Scenedesmus* sp.

During the characterization of isolated PHA, the FT-IR spectrum showed prominent peaks at 1746 and 1160 cm^−1^ (Figure 3). These peaks denote a carbonyl group and stretching vibration of asymmetric C–O–C, respectively, both characteristic for the ester bond found in PHA molecules. Other absorption bands obtained at 1374, 1460, and 2952 cm^−1^ denote groups –CH_3_, –CH_2_, and –CH, respectively. The absorption bands at 1023–1094 cm^−1^ were attributed to stretching vibrations of C–O, which can be due to amorph PHB. Almost identical peaks denoting various PHA functional groups were observed on samples for the rest of the experiments (See Appendix A).

### 3.3. Experiment Design and Evaluation of the Relevant Factors

The number of variables that could potentially affect PHB production was relatively high. Five factors, including glucose, nitrogen, phosphorus, iron, and salinity levels, were considered, in principle, influential. A full two-level factorial design (2^5^) would involve a total of 32 experiments, plus the replicates necessary for the evaluation of the degree of coincidence between the results. Therefore, a two-level Taguchi design involving 16 random runs was selected. Table 2 shows the design matrix for the experiment and the total production yield of PHB.

**Figure 4:**
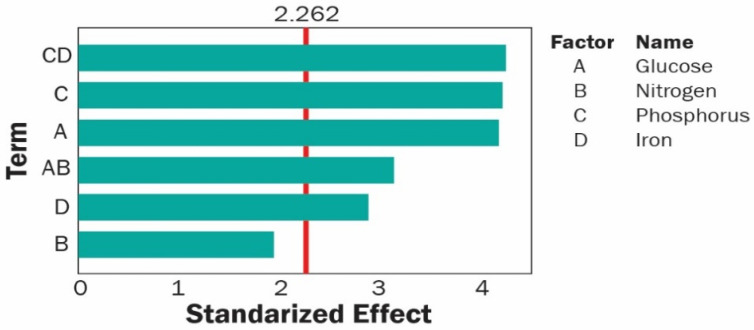
Pareto chart for the standardized main effects (after discarding effect E, salinity) and interactions effects between variable pairs. Response is PHA concentration. The vertical line indicates the statistical significance bound for the effects. For this experiment, factors with a *p*-value < 0.05 were considered significant.

Analysis of the results given in the last column of Table 2 using Minitab 16 allowed discarding the less significant factors and interactions. For this experiment, factors with a *p*-value < 0.05 were considered significant. The first analysis of the data allowed salinity to be excluded as a relevant factor, and with this consideration, the relevant parameters were identified. The remaining four factors and their interactions were then analyzed and after iterative evaluations, we obtained the more restrictive model presented on the Pareto chart of Figure 4. Based on their *p*-values, glucose, phosphorus, iron, and the glucose-nitrogen and phosphorus-iron interactions were identified as relevant factors for PHA production by *Scenedesmus* sp. The tests for normality, constant variance, and independence of the residuals of the response observations yielded favorable results (See Appendix A).

The ANOVA analysis allowed us to obtain the coefficients of each relevant factor with which the equation that models the PHA production was constructed. Discarding the insignificant factors, the model equation for PHA can be written as:

$$Y_{PHA}\;=\;12.39\;-\;0.57A\;+\;0.30B\;+\;0.26C\;+\;554.22D\;-\;0.19AB\;-\;2873.83CD$$

where *A*, *B*, *C*, and *D*, are glucose, nitrogen, phosphorus, and iron concentrations, respectively. The quality of the model equation is expressed by the coefficient R^2^, which has a value of 0.89, indicating a good quality fit.

Using Minitab’s response optimizer tool, we determined which are the levels of each factor that favor bioplastic production and obtained a prediction of the maximum expected production when those levels are used, which is 25.92% *w*/*w* DW.

### 3.4. Influence of Relevant Factors on the Bioproduction of PHA

In this study, the normal growth condition yielded a production of 8.61% (*w*/*w* DW) in *Scenedesmus*. Adapting the cells to phosphate deficient media (with a low level of glucose) increased the production level to 29.92%. Under heterotrophic growth (high glucose) and normal nutrients concentration, accumulation of PHA was 2.96%. This time, P-depletion resulted in a 3.6-fold increase on the production of biopolymer (10.75%). It was followed by increase of PHA production as an effect of other parameters.

*Scenedesmus* sp. grown under iron limitation and low glucose accumulated 12.2% of PHA. Under heterotrophic growth with Fe-depletion 2.27% of PHA was present, a slightly smaller concentration of that obtained without iron restriction (2.96%).

With a low level of glucose, the iron-depleted growth condition together with normal phosphorus concentration yielded an accumulation of 12.2% (*w*/*w* DW) of PHA in *Scenedesmus* sp. Adapting the cells to both iron and phosphorus-deficient media with the same glucose level resulted in a similar production of 13.8% (*w*/*w* DW). However, when *Scenedesmus* grew with normal iron concentration, PHA production increased from 8.61 to 29.92% *w*/*w* DW when phosphorus was absent from the media, the highest concentration obtained on our experiments.

For *Scenedesmus* sp. under heterotrophic growth, the effect of the iron-phosphorus interaction over PHA production was similar. When iron was absent from the medium, the PHA production from *Scenedesmus* sp. remained at low levels for both high and low phosphorus conditions (2.27 and 0.82% *w*/*w* DW of PHA, respectively). This last condition (iron and phosphorus omission with glucose addition) yielded the lowest PHA production obtained on these experiments (0.82% *w*/*w* DW). However, when iron was present in the culture, phosphorus absence increased PHA production from 2.96 to 10.75% (*w*/*w* DW).

As mentioned previously, under normal growth conditions with low addition of glucose, *Scenedesmus* sp. reached a production of 8.61% (*w*/*w* DW) of PHA. When the 4 g L^−1^ of glucose was added to the medium without nutrient restriction, PHA production decreased to 2.96% (*w*/*w* DW).

When the glucose addition was combined with nutrient limitation, we observed overall decrements on PHA production. Glucose addition caused a small decrease (from 9.07 to 8.13% *w/w* DW) in the accumulation of PHA on experiments whose only restriction was nitrogen deficiency. A bigger drop was observed when glucose was added to the cultures that were only phosphate restricted. This time PHA production fell from 29.92% to 10.75 *w*/*w* DW, almost a 3-fold lower production. Supplementing iron-deficient cultures of *Scenedesmus* sp. with the external carbon source also decreased their accumulation of the storage compound (from 12.2 to 10.75% *w*/*w* DW).

As stated previously, under nitrogen deprived conditions, PHA production variated just slightly with both of the glucose levels considered (8.13 and 9.07% *w*/*w* DW for the high and low levels, respectively). However, when BG-11 nitrogenated compounds were present in the culture medium, glucose level notably influenced net PHA yield. PHA production by *Scenedesmus* sp. cultured without nitrogen restriction was almost 3-fold higher with low glucose than that with high glucose concentration (8.91 and 2.96% and *w*/*w* DW).

### 3.5. Accumulation of Lipids and Carbohydrates

In addition to PHA’s accumulation, a wide variation on lipids and carbohydrates contents measurements were found depending on the culture conditions and growth stage of each experiment in considerable yield amounts. For both compounds, there was increased accumulation towards day 14 of cultivation. Cultures 1 and 9 had the highest lipid content at the end of the experiment with 15.4 and 15.46% of the dry weight, respectively. Culture 6 presented the lowest lipid accumulation, with only 2.49%. Carbohydrates were detected in concentrations as high as 24.59 and 28% in cultures 1 and 2, respectively. The minimum was 3.42% for culture 16.

This section may be divided by subheadings. It should provide a concise and precise description of the experimental results, their interpretation as well as the experimental conclusions that can be drawn.

## 4. Discussion

Since the first finding of PHA accumulation in cyanobacteria, a number of studies have reported its occurrence in various microalgae strains. However, to our better knowledge, PHA production by *Scenedesmus* sp. had not been documented elsewhere, nor the parameters that affect their accumulation were known. In this study, out of the 16 different experiments performed with *Scenedesmus* sp., we detected PHA accumulation in all of them, with concentrations ranging from 0.82 to 29.92% *w*/*w* CDW. Higher PHA (29.92%) was detected in experiment 6 (P-deficiency, normal N and Fe, low glucose, low salinity). Similar values were reported for *Nostoc muscorum* (31%) supplemented with acetate and propionate [77] and *Spirulina* sp. (30.7%) under nitrogen deficiency [78]. It is notable that this value was higher than those that have been obtained under optimized conditions for *Botryococcus braunii* (16.4%) [79] and *Synechocystis* sp. (11%) [80]. *Scenedesmus* sp. cultivated without nutrient deficiency on standard BG-11 medium accumulated 8.61% of PHA. Lower productions of about five percent were reported for *Synechocystis* sp. [68] and *Gloeocapsa gelatinosa* [53] growth in the same medium conditions.

Our study explored the effect of four stress conditions: N, P, and Fe limitation, and NaCl addition; and of glucose supplementation, on the accumulation of PHA by *Scenedesmus* sp. To analyze all these variables simultaneously, we carried out a fractional Taguchi experimental design. This balanced design makes use of orthogonal arrangements so that all the factor levels are weighted equally. Thus, we were able to evaluate each factor independently of the rest by carrying out a reduced number of experiments. After performing the experiments and statistical analysis of the response, we determined that the levels of phosphorus, glucose, and iron influenced the production of PHA by *Scenedesmus* sp., and that the glucose-nitrogen and phosphorus-iron interactions were also significant.

**Figure 5:**
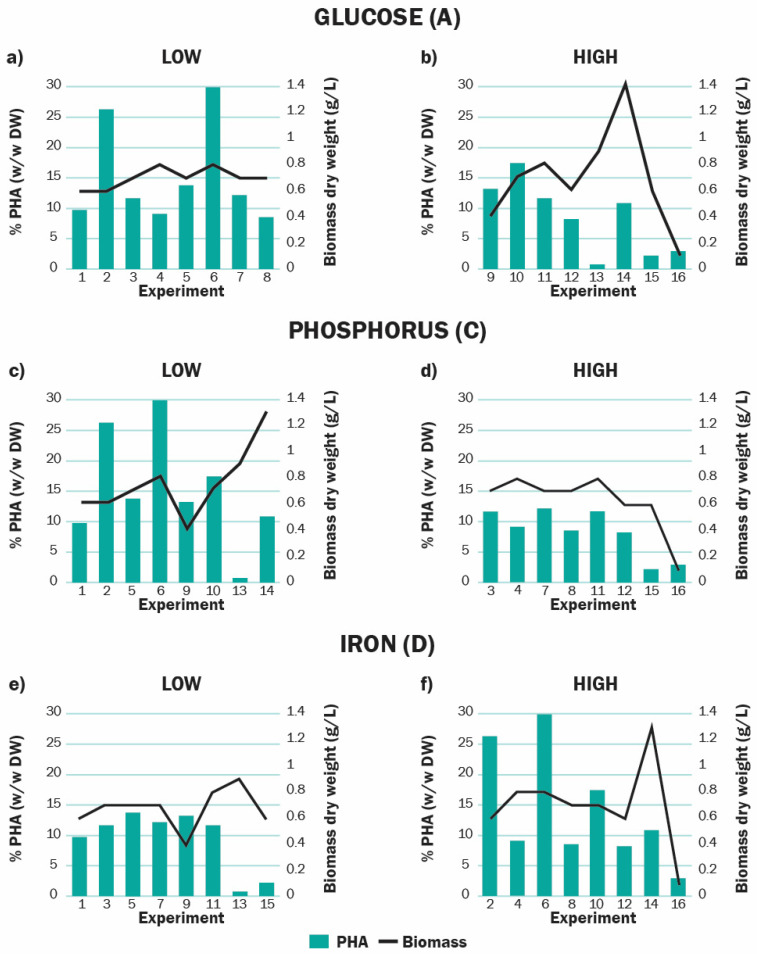
Analysis of the effect of the main significant factor over the production of *Scenedesmus* sp. at day 14 with respect to PHA (%) and biomass (dry weight). The yield obtained with the low and high levels of glucose (**a**,**b**), phosphorus (**c**,**d**), and iron (**e**,**f**) is shown.

Phosphorus deficiency enhanced PHA accumulation on the 14th day compared to cultures where P was present in the medium (Figure 5c,d). This goes under the observations made in other works where phosphate limitation has also induced increased bioplastic accumulation [49,51,53,81,82,83]. It has been proposed that phosphorus restriction can raise PHA levels by increasing the number of enzymes in the PHA synthetic pathway. Phosphorus deficiency decreases the size of the total ATP pool [84], but the reduction of NADP to yield NADPH continues. This increases the intracellular concentration of NADPH and stimulates PHA synthesis while inhibiting citrate synthase activity in the TCA cycle [85], which further promotes PHA accumulation by ensuring the availability of acetyl-CoA for β-ketothiolase.

Iron is an important biochemical component necessary for cell growth, chlorophyll production, and nitrogen metabolism [56]. Previously, Rizwan et al. [86] found the presence of this element enhances the accumulation of energy storage compounds such as carbohydrates and lipids in marine microalgae *Dunaliella tertiolecta*. However, little work has been done investigating the role of this trace metal on the biosynthesis of PHA by microalgae, although it is certainly involved in many of their metabolic pathways. Our results indicate that iron presence is relevant for PHA production, and specifically, that Fe depletion decreases bioplastic accumulation (Figure 5e,f). Previous studies [87] found that iron-limited cultures of *Scenedesmus Quadricauda* direct twice as much of the total carbon fixed into protein formation compared to non-Fe limited cultures. This could explain the diminished PHA productivity of iron-limited cultures since the substrate carbon is probably directed towards protein fixation processes rather than being accumulated as reserve carbon. Thus, iron presence is crucial to avoid the deviation of the metabolic activity of *Scenedesmus* sp. away from PHA production.

**Figure 6:**
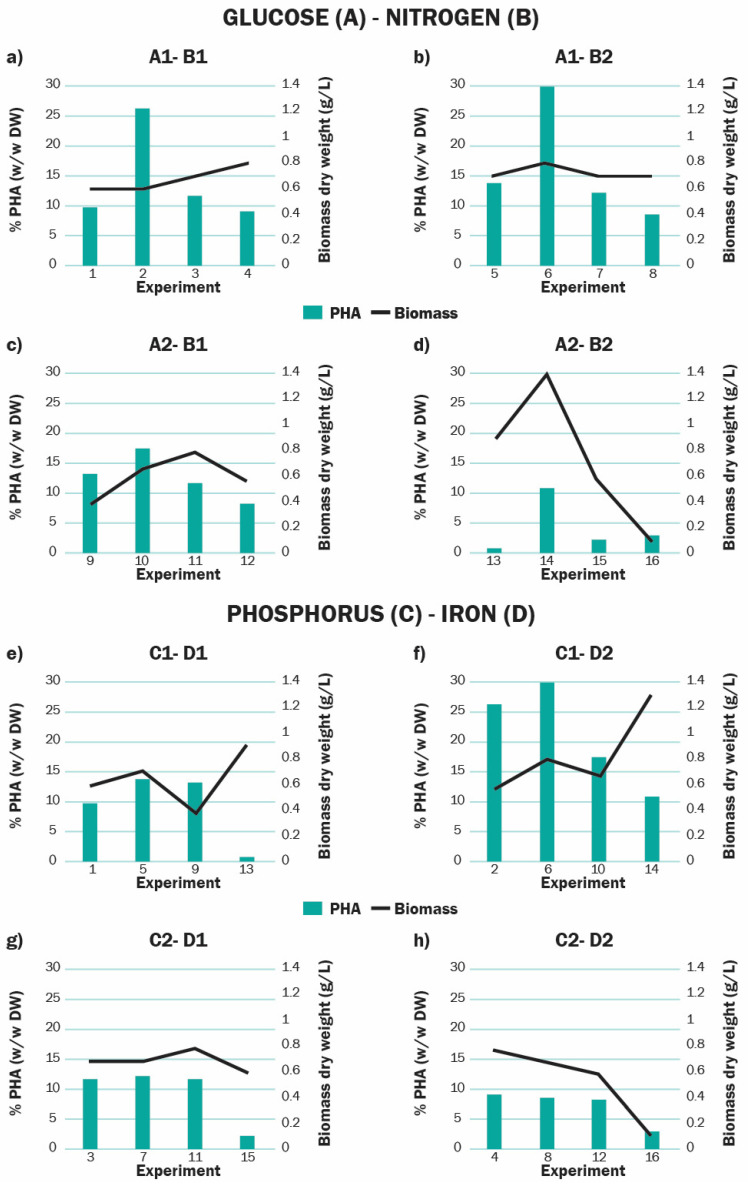
Analysis of the effect of significant factor interactions over the production of *Scenedesmus* sp. at day 14 with respect to PHA (%) and biomass (dry weight). The yield obtained with each level combination of glucose-nitrogen (**a**–**d**) and phosphorus-iron (**e**–**h**) is shown.

We also found that although phosphorus limitation enhances PHA accumulation by *Scenedesmus* sp., iron needs to be also present on the medium to achieve a higher concentration of the product (Figure 6e–h). This Fe-P interaction is statistically significant and could be related to the Fe role in P acquisition under P-limited conditions. When microorganisms grow in a P-limited medium, they can access part of the dissolved phosphorus via alkaline phosphatase enzymes (APases). These enzymes are also involved in the utilization of intracellular P reservoirs or of other P-containing intracellular compounds [88]. Recently, Browning et al. [61] found that iron is an important cofactor on the upregulation of these enzymes by marine microorganisms. They found that the presence of Fe on P-limited cultures induces an increase in the activity of APases. This suggests that there is a biological dependence of extracellular P acquisition and intracellular P utilization on Fe availability. Iron could then be necessary for the microalgae to access sufficient P to follow the metabolic route for PHA accumulation, since prolonged phosphate deficiency limits fundamental physiological processes, which indirectly stops PHA synthesis [53].

It is known that nutritional mode can markedly influence biomass growth and productivity of microalgae [89]. In this study, cells that were grown in medium with high supplementation of glucose produced significantly less accumulation of PHA on day 14 (Figure 5a,b). Although it has been reported that addition of low concentrations of external organic carbon enhances PHA production by cyanobacteria [52,53], in this case the excess of organic carbon was not assimilated in the form of PHA, but probably contributed to biomass accumulation. When analyzing biomass growth (Figure 2), it can be seen that for the sixteen experiments, the cultures did not present the lag phase of adaptation, instead beginning in the exponential phase. This was probably because inoculum was composed of exponentially growing cells from preculture. The addition of a high amount of glucose could have served to sustain this exponential growth instead of helping cultures to enter the stationary phase. It has been reported that PHA starts to accumulate at the end of the exponential growth [52,90], so prolonging this phase probably retarded the biosynthesis of PHA, reducing its overall productivity. Our findings on the effect of glucose addition over the PHA production represent an advantage for the use of *Scenedesmus* as a producing organism at industrial levels. Since there is no requirement for a large amount of exogenous carbon supplementation, the fermentative production of PHA by this strain would be less expensive than that by other bacteria and microalgae strains.

Of the numerous studies searching to enhance PHA production by means of imposing nutritional stress on microalgae, the vast majority reported that nitrogen deprivation usually promotes higher bioplastic accumulation [49,52,53,54]. In this study, nitrogen limitation alone did not significantly enhance PHA productivity for *Scenedesmus* sp. This could indicate that N depletion does not alter the physiological balance of *Scenedesmus* enough to trigger the bioproduction of PHA as a reservoir compound. In fact, it has been demonstrated that *Scenedesmus* sp. can grow under different levels of N-limitation [91].

The interaction of glucose and N was found relevant for PHA accumulation, although the individual impact of N concentration was not significant. Cells grown with simultaneous supplementation of N and a high level of glucose registered a drastic fall in PHA accumulation (Figure 6a–d). This was probably because under this condition, biomass growth was stimulated rather than synthesis of storage compounds, as it has been reported for other microalgae strains [92]. The fact that, in our experiments, cultures under this condition (13, 14, 15, 16) had some of the highest biomass productivities (0.1670 and 0.1469 g L^−1^d^−1^ on day 7 for experiments 13 and 14, and 0.1172 and 0.1047 g L^−1^d^−1^ on day 4 for experiment 15 and 16), also support this statement. Devi et al. [89] also observed this trend in their study with nine microalgae strains, where C (as organic carbon) and N supplementation resulted in higher biomass and pigments production. If that was the case, *Scenedesmus* sp. in the experiments 13, 14, 15, and 16 could have assimilated those two nutrients in the form of biomass and proteins, leading to an adverse PHA production. However, this condition could be useful if a two-stage cultivation approach is utilized, as it can help to achieve high concentrations of biomass on a first growth phase, previous to a starvation phase when PHA production would be stimulated by restricting *Scenedesmus* sp. access to nutrients in a controlled fashion. Regarding the growth process, it is difficult to speed up this parameter since growth time depends on the microalgae strain. However, as it can be seen on Figure 2, under specific nutritional conditions, microalgal cells entered the stationary phase faster (on days 7, 10, or 13). By that time, PHA already started to accumulate (in the previous exponential phase) on those cultures. Given that Scenedesmus can enter faster to the production of PHA, it can be designed as a process where time optimization is used as fed-batch.

Any significant effect on PHA production was not observed when different concentrations of NaCl were added to the medium. This result contrasts with the works of Ansari and Fatma [53] with *N. muscorum* and Shrivastav et al. [93] with *Spirulina subsalsa*, both freshwater strains that showed enhanced PHA accumulation under salinity stress. This could be due to the fact that, although *Scenedesmus* sp. is also considered a freshwater strain, it has shown great tolerance to salinity on the medium over a wide range of NaCl concentrations [94].

The production of bioplastic from microalgae has still not been developed at commercial scale since the overall economical balance is still higher than that of traditional plastics and other polymeric materials. The cost associated with the extraction and purification of the biopolymer must be considered, as it can represent up to 50% of the total process cost [95]. The environmental impact of the reagents used is also of importance. The extraction of PHA usually involves the use of solvents such as chloroform, which although being the one that results in better PHA recuperation rates yields and can be recovered and potentially recirculated to be reused in subsequent extractions, it is also toxic and represents environmental hazards. A number of authors have explored the use of non-halogenated organic solvents such as ethylene carbonate [96] and some called poor-solvents (acetone and ethanol) [97] at high temperatures (above 100 and 150 °C) for PHA extraction achieving yields comparable to that obtained using chloroform. This solvents could be more cost-efficient, while being also recyclable and environmental friendly.

Since microalgae produces many components with considerable market value, it has been proposed that the industrial production of PHA may be feasible when it is synthetized along with other high value compounds [98,99]. Microalgae strains have been usually studied for the obtention of individual bioproducts, but a biorefinery for the production of multiple substrates for different industries such as bioenergy and biomaterials (bioplastics) could result in being more cost effective. In this study, *Scenedesmus* was capable of accumulating PHA along with lipids and carbohydrates, which can serve for biodiesel and bioethanol production. Previous studies have explored the use of *Scenedesmus* for biorefinery development and have found that when performed in an optimal sequence, extraction of multiple metabolites could provide net value gains of around 66% over the potential cost of microalgae cultivation [100]. Thus, an adequate consideration of each one of the processing stages, their yields and the market value of the obtained products is necessary for the process to be economically viable.

## 5. Conclusions

*Scenedesmus* sp. is a freshwater microalgae strain that has been previously studied for biogas fixation, removal of nutrients from waste streams, production of lipids and carbohydrates. This work reported for the first time the production of PHA by *Scenedesmus* sp. and identified the nutritional factors that influence its accumulation as a crucial step for the exploitation of this strain as a biopolymer producer organism. Phosphorus, glucose, and iron together with the glucose-nitrogen and phosphorus-iron interactions were statistically significant for induction of PHA accumulation. The wide variation obtained among the produced bioplastic (0.83–29.92%, *w*/*w* DW) reflects the importance of the medium conditions during cultivation to achieve a high accumulation of the biopolymer. In addition to a base accumulation of 8.61% of PHA under normal growth conditions (higher than that of previously studied microalgae strains), there was about a 3.4-fold rise in PHA accumulation in *Scenedesmus* sp. only by manipulating the levels of basic nutrients in the culture. *Scenedesmus* sp. do not require supplementation with large amounts of exogenous carbon to produce PHA, an economical advantage over the use of higher accumulating bacteria. Also, its tolerance to salinity stress could make its cultivation possible using water with a certain concentration of NaCl, instead of drinking water, which is a non-renewable resource. Finally, we detected co-accumulation of lipids and carbohydrates, macromolecules of interest for the production of biofuels and bioethanol, on all the experiments. For these reasons, *Scenedesmus* sp. stands out as a potential candidate to produce polyhydroxyalkanoates (a promising biodegradable and biocompatible bioplastic) and other useful macromolecules. Further studies to optimize the co-accumulation of these three major compounds would create a means for maximal utilization of *Scenedesmus* sp. bioresources.

## Figures and Tables

**Table 1:** Factor levels on Taguchi design.

Factors		Levels	
Variable	Name	Low (1)	High (2)
Glucose (g L^−1^)	A	1	4
Nitrogen (mM)	B	0	17.6
Phosphorus (mM)	C	0	0.23
Iron (Mm)	D	0	0.21
Salinity (g L^−1^)	E	0.5	2

**Table 2:** Design matrix response values for Taguchi design.

Run	Glucose (g L^−1^)	Nitrogen (mM)	Phosphorus (mM)	Iron (mM)	Salinity (g L^−1^)	Polyhydroxyalkanoate (PHA) (% *w*/*w*)	PHA (g L^−1^)
1	1	0	0	0	0.5	9.793	0.064
2	1	0	0	0.021	2	26.25	0.171
3	1	0	0.23	0	0.5	11.68	0.082
4	1	0	0.23	0.021	2	9.075	0.073
5	1	17.6	0	0	2	13.80	0.104
6	1	17.6	0	0.021	0.5	29.92	0.239
7	1	17.6	0.23	0	2	12.20	0.085
8	1	17.6	0.23	0.021	0.5	8.612	0.060
9	4	0	0	0	2	13.08	0.052
10	4	0	0	0.021	0.5	17.14	0.120
11	4	0	0.23	0	2	11.60	0.087
12	4	0	0.23	0.021	0.5	8.135	0.049
13	4	17.6	0	0	0.5	0.831	0.007
14	4	17.6	0	0.021	2	10.75	0.134
15	4	17.6	0.23	0	0.5	2.267	0.014
16	4	17.6	0.23	0.021	2	2.959	0.030

**Figure A1:**
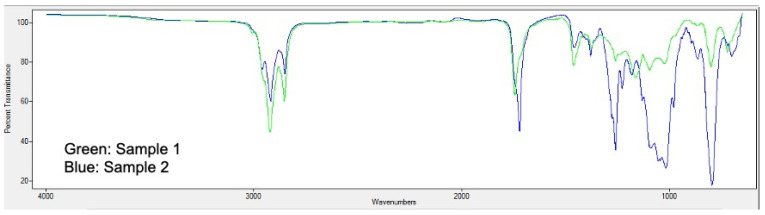
FI-IR spectrum of isolated PHA from experiments 1 and 2 with *Scenedesmus* sp.

**Figure A2:**
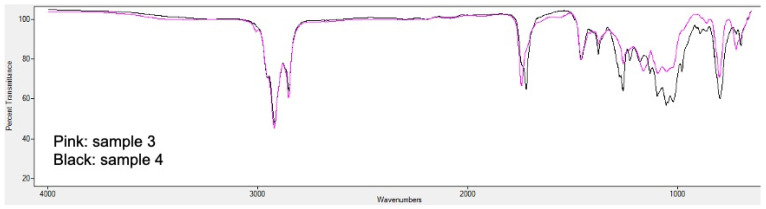
FI-IR spectrum of isolated PHA from experiments 3 and 4 with *Scenedesmus* sp.

**Figure A3:**
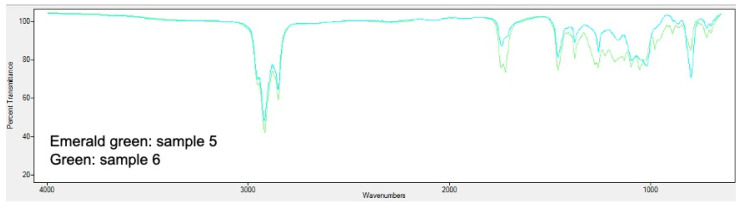
FI-IR spectrum of isolated PHA from experiments 5 and 6 with *Scenedesmus* sp.

**Figure A4:**
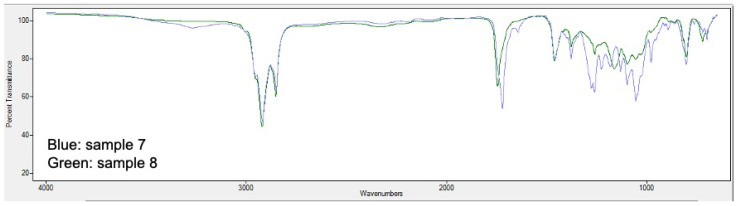
FI-IR spectrum of isolated PHA from experiments 7 and 8 with *Scenedesmus* sp.

**Figure A5:**
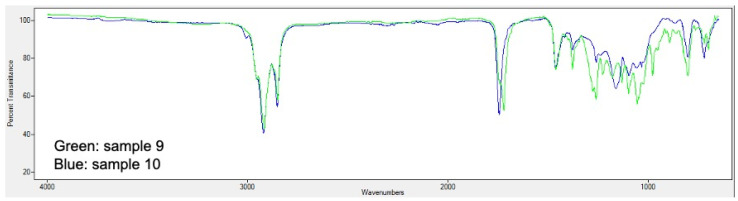
FI-IR spectrum of isolated PHA from experiments 9 and 10 with *Scenedesmus* sp.

**Figure A6:**
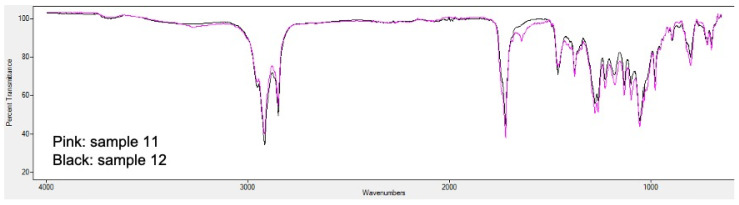
FI-IR spectrum of isolated PHA from experiments 11 and 12 with *Scenedesmus* sp.

**Figure A7:**
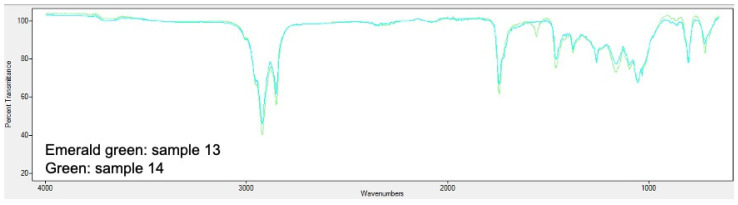
FI-IR spectrum of isolated PHA from experiments 13 and 14 with *Scenedesmus* sp.

**Figure A8:**
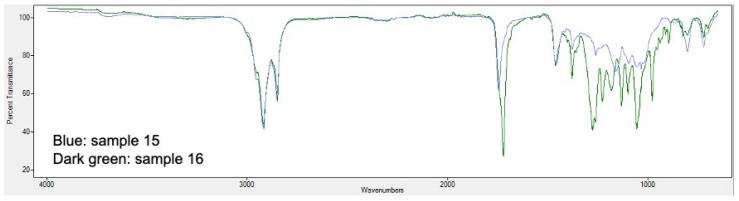
FI-IR spectrum of isolated PHA from experiments 15 and 16 with *Scenedesmus* sp.

**Figure A9:**
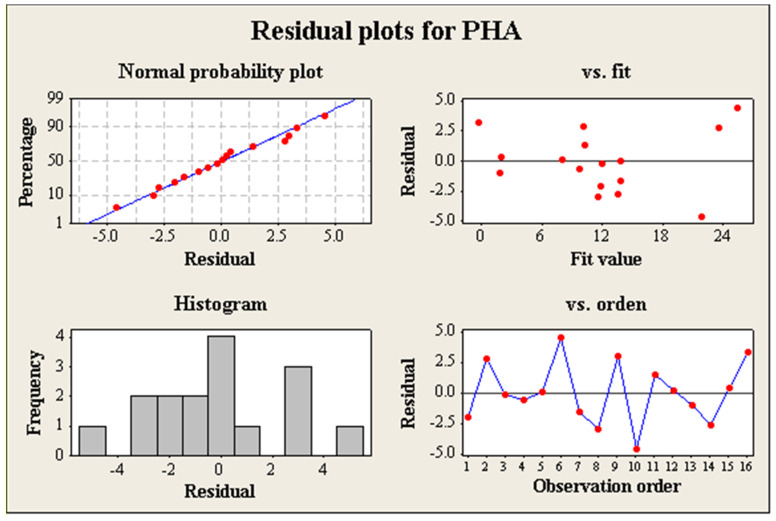
Test to check for the normality, constant variance and independency of the residuals from the ANOVA analysis.

**Figure A10:**
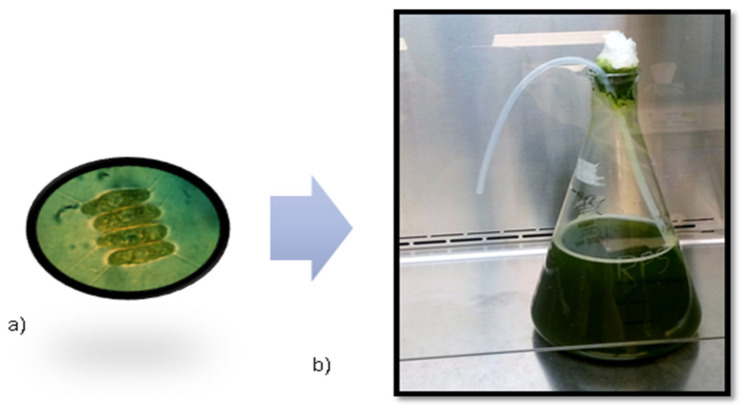
*Scenedesmus* sp. obtained from the UTEX culture collection (**a**) microscope view (**b**) culture used as inoculum.

**Figure A11:**
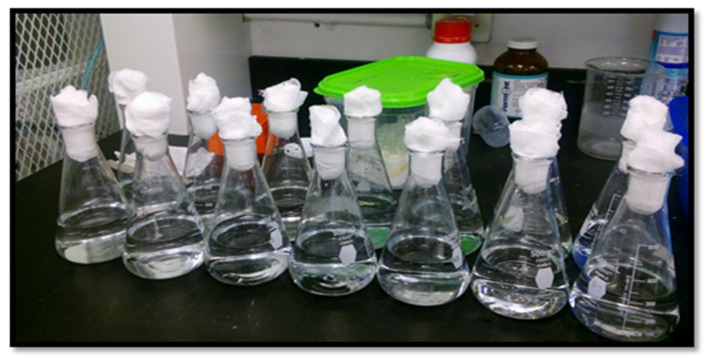
Sixteen different culture media prepared for the experiment.

**Figure A12:**
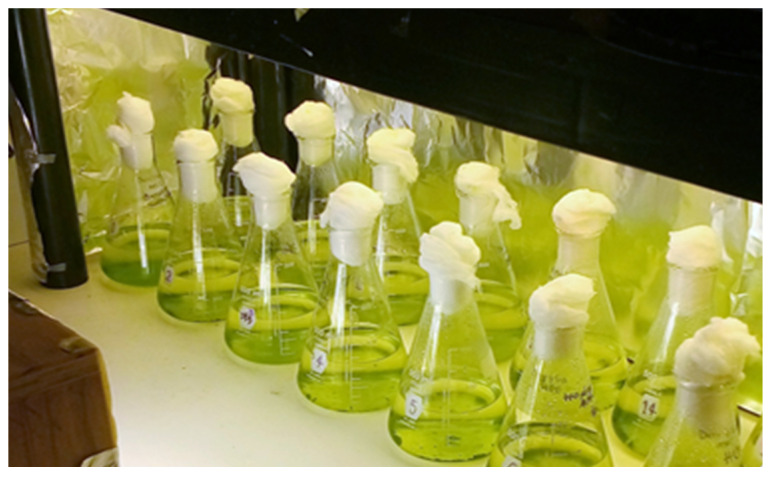
Cultivation of *Scenedesmus* sp. under diverse nutritional conditions.

**Figure A13:**
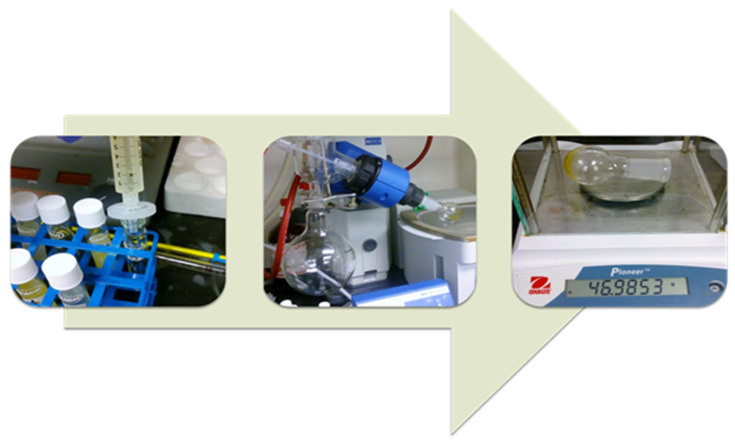
Some phases of the PHA extraction process.

## Data Availability

Not applicable.
